# Cognitive appraisals of dissociation in psychosis: a new brief measure

**DOI:** 10.1017/S1352465820000958

**Published:** 2021-07

**Authors:** Emma Černis, Jessica C. Bird, Andrew Molodynski, Anke Ehlers, Daniel Freeman

**Affiliations:** 1 Oxford Cognitive Approaches to Psychosis, Department of Psychiatry, University of Oxford, Oxford, UK; 2 Oxford Health NHS Foundation Trust, Oxford, UK; 3 Oxford Centre for Anxiety Disorders and Trauma, Department of Experimental Psychology, University of Oxford, Oxford, UK

**Keywords:** cognitive appraisals, dissociation, hallucinations, paranoia, psychosis

## Abstract

**Background:**

Catastrophic cognitive appraisals, similar to those in anxiety disorders, are implicated in depersonalisation, a form of dissociation. No scales exist to measure appraisals of dissociative experiences. Dissociation is common in psychosis. Misinterpretations of dissociative experiences may maintain psychotic symptoms. Therefore, assessing appraisals in this context may be valuable.

**Aims:**

The primary aim was to develop a measure of key appraisals of dissociation in psychosis. Secondary aims were to test the relationship between appraisals and psychotic experiences (paranoia and hallucinations), and determine whether appraisals explain additional variance in psychotic symptoms above dissociative symptoms.

**Method:**

Fifty items were generated from transcripts of interviews with patients. The measure was developed and psychometrically validated via factor analysis of data from 9902 general population participants and 1026 patients with psychosis. Convergent validity, test–re-test reliability, and internal reliability were assessed. Regression analyses tested relationships with psychotic symptoms.

**Results:**

A 13-item single-factor measure was developed. Factor analysis indicated good model fit [χ^2^(65) = 247.173, comparative fit index (CFI) = 0.960, root mean square error of approximation (RMSEA) = 0.052]. The scale had good convergent validity with a rumination (non-clinical: *r* = 0.71; clinical: *r* = 0.73) and dissociation measure (*r* = 0.81; *r* = 0.80), high internal consistency (α = 0.93; α = 0.93), and excellent 1-week test–re-test reliability [intraclass correlation (ICC) = 0.90]. It explained variance in psychotic symptoms (paranoia: 36.4%; hallucinations: 35.0%), including additional variance compared with dissociation alone (paranoia: 5.3%; hallucinations: 2.3%).

**Conclusions:**

The Cognitive Appraisals of Dissociation in Psychosis (CAD-P) measure is a psychometrically robust scale identifying appraisals of dissociative experiences in psychosis and is associated with the presence of psychotic experiences. It is likely to prove useful for clinical assessment and research.

## Introduction

Dissociation is characterised by experiences of disconnection or alteration, such as feeling detached from one’s own thoughts and emotions, finding one’s own reflection unfamiliar, or seeing the world as ‘flat’ or unreal (Kennedy *et al*., 2013). Increasingly, dissociative experiences are being recognised as important transdiagnostic phenomena, occurring in diagnoses other than post-traumatic stress disorder (PTSD) (Lyssenko *et al*., 2018), and as important clinical phenomena in their own right. For example, research is demonstrating how such experiences are independently associated with distress and increased self-harm (Černis *et al*., 2019) and suicidality (Calati *et al*., 2017). However, dissociation remains under-recognised by clinicians (Bailey and Brand, 2017).

Hunter and colleagues (2003) outline a cognitive model for the dissociative diagnosis of depersonalisation disorder (DPD), and Baker and colleagues (Baker *et al*., 2007) propose a model for the dissociative experiences of ‘depersonalisation and feelings of unreality’. Both models highlight the role of ‘catastrophic attributions’ for the dissociative experience as central to the occurrence of the disorder, in a manner similar to that found in anxiety disorders, and particularly panic disorder (Clark, 1999). As in panic disorder, Hunter and colleagues propose that the transient symptoms experienced in DPD are misinterpreted as indicative of impending catastrophe. In panic disorder, this may be heart palpitations being mistaken as a sign of imminent heart attack; whilst in DPD a feeling of detachment may be taken as a sign of becoming ‘mad’. Cognitive appraisals of dissociative symptoms as dangerous therefore result in an increase in anxiety (further exacerbating the dissociative symptoms), and lead to behaviours intended to neutralise the threat, but which actually serve to heighten attention towards them – such as monitoring symptoms and increasing introspection (Hunter *et al*., 2003). In the course of empirically testing this model, Hunter *et al*. (2014) demonstrated that a DPD group were less likely than a non-clinical group to make normalising attributions when prompted to generate as many reasons in one minute why they might experience various DPD, anxiety and neutral symptoms. In a second task, the DPD group were found to endorse catastrophic appraisals about mental illness or brain dysfunction in a manner similar to an anxiety disorder (obsessive compulsive disorder or panic disorder) comparison group. Both these findings support the hypothesis that anxiety-like cognitive appraisals may be important in this form of dissociation: however, they do not identify cognitive appraisals that are specific to dissociative experiences.

Dissociation may be particularly common in psychosis (Renard *et al*., 2017). Based on patient reports, we have characterised dissociation in this context as taking the form of an unanticipated subjective experience of anomaly (felt sense of anomaly, FSA), which may include experiences of disconnection, unfamiliarity and unreality (Černis *et al*., 2020). Consideration of dissociation may be important in psychosis research given the ‘robust and well-replicated’ associations between dissociation and psychotic symptoms (Longden *et al*., 2020), and suggestions that anomalous experiences – such as dissociation – may contribute to the development and maintenance of psychotic symptoms (Freeman, 2016; Garety *et al*., 2001). Consistent with the depersonalisation literature, anxiety processes have also been implicated in dissociation in this context (Černis *et al*., 2014; Freeman *et al*., 2013). However, to date, there has been no research into the cognitive appraisals that may be important in the maintenance of dissociation.

Clinicians can elicit in assessment appraisals of dissociation, but this process can be greatly aided by a questionnaire of key appraisals tested in large populations. It is also the case that before research can investigate the role of cognitive appraisals, a method for eliciting and measuring them is required. The objective of this study, therefore, was to develop a measure of key cognitive appraisals of dissociative experiences arising in the context of psychosis to aid clinicians and researchers in the detection, assessment and treatment of dissociation.

## Part 1: Development of the measure with a general population sample

### Method

#### Procedure and participants

The design was an online cross-sectional self-report questionnaire study. Participants were recruited via social media, the majority via Facebook advertisements. The advertisements were titled ‘Understanding Dissociative Experiences’ and stated that researchers were seeking participants to ‘to complete questionnaires about different kinds of thoughts’, and that they need not have experienced dissociation to take part. The information sheet described dissociation as a range of experiences ‘where people describe feeling “strange” or “disconnected” – they might feel like they are “spacing out”, feel “unreal” or feel emotionally detached from the world’. Inclusion criteria were deliberately broad: any adult (age 18 years or over), usually resident in the United Kingdom (UK). There were no exclusion criteria.

All responses were collected via online surveys using Qualtrics (2018). The survey landing page contained the participant information sheet and statements regarding informed consent (British Psychological Society, 2017). Surveys were accessible on desktop and mobile web browsers. Incomplete surveys were retrieved automatically after a week of non-activity and added to the dataset.

There were three phases of data collection. Data collection for Phase 1 (full item pool) ran from 11 to 21 January 2019; and for Phase 2 (refined item pool) from 30 January to 26 February 2019. Phase 3 (test–re-test) data were collected from a subsample of Phase 2 participants between 5 and 23 April 2019. Following the removal of cases with high levels of missing data in the item pool (greater than 20% missing), sample sizes for the three phases were *n* = 1615, *n* = 8287 and *n* = 140, respectively. For all samples, the majority of participants were White (>94.00%) and female (≥80.00%). The mean ages of the samples were 49.94 (*SD* = 14.40), 45.84 (*SD* = 14.86) and 46.69 years (*SD* = 15.27), respectively. See Table 1 for full demographic details.

**Table 1.** Demographic data and descriptive statistics for all groups

Participant group		Phase 1	Phase 2	Test–re-test (subset of Phase 2)	Clinical
**N**		1615	8287	140	1026
**Age** Mean (*SD*)		49.94 (14.40)	45.84 (14.86)	46.69 (15.27)	41.49 (12.29)
**Gender** *n* (%)	Female: Male: Other:	1433 (88.73%) 142 (8.79%) 35 (2.17%)	7103 (85.71%) 962 (11.61%) 165 (1.99%)	112 (80.00%) 20 (14.29%) 6 (4.29%)	300 (29.24%) 717 (69.88%) 5 (0.49%)
**Ethnicity** *n* (%)	White (any): Mixed/multiple: Asian (any): Black (any): Other:	1551 (96.04%) 25 (1.55%) 12 (0.74%) 3 (0.19%) 6 (0.37%)	7866 (94.92%) 175 (2.11%) 80 (0.97%) 20 (0.24%) 38 (0.46%)	133 (95.00%) 3 (2.14%) 2 (1.43%) 0 (0%) 0 (0%)	687 (66.96%) 43 (4.19%) 98 (9.55%) 175 (17.06%) 17 (1.66%)
**Measure** Mean (*SD*)	CAD-P	19.05* (10.70)	16.58 (11.79)	Week 1: 20.12 (10.69) Week 2: 19.87 (11.17)	18.70 (13.35)
	ČEFSA	—	41.76 (26.14)	—	39.65 (30.51)
	PTQ	—	34.97 (13.83)	—	30.32 (16.38)
**Have you ever experienced mental health difficulties?** *n* (%)	Yes No	1222 (75.67%) 360 (22.29%)	6584 (79.45%) 1557 (18.79%)	—	—
**If ‘yes’, are these ongoing?** *n* (%)	Yes No	886 (72.50%) 312 (25.53%)	4919 (74.71%) 1520 (23.09%)	—	—
**Diagnosis** *n* (%)	Schizophrenia Schizoaffective Delusional disorder Psychotic disorder NOS** First episode psychosis Other SSD	—	—	—	662 (64.52%) 153 (14.91%) 14 (1.36%) 69 (6.73%) 105 (10.23%) 23 (2.24%)

CAD-P Cognitive Appraisals of Dissociation in Psychosis

ČEFSA Černis Felt Sense of Anomaly scale

PTQ Perseverative Thinking Questionnaire

SSD Schizophrenia Spectrum Disorder

* *Contains item ‘I’m so lonely’ which was later amended to ‘I am all alone’*

** *Not otherwise specified (NOS) also includes unspecified non-organic psychosis*

#### Measures

Fifty items about cognitive appraisals of dissociation (e.g. ‘this might last forever’) were developed by the lead author from transcripts of interviews carried out for a qualitative study (Černis *et al*., 2020). In this qualitative study, 12 NHS patients with psychosis diagnoses and experience of dissociation were asked directly what thoughts they had in response to dissociation. Nine of the 12 patients scored within the range expected for dissociative disorders on the Dissociative Experiences Scale (Carlson and Putnam, 1993), with the mean score of 36.91 (*SD* = 20.00) indicating high levels of currently experienced dissociation within the group (Černis *et al*., 2020).

Participants in the online study rated the 50 items (see Supplementary material) for the past two weeks on a Likert scale from ‘0, never’ to ‘4, always’ with the instruction ‘please rate how often you think the following when you are feeling strange, disconnected, unreal or “dissociated”’. Participants were prompted to answer to the best of their ability, and only to rate sensations that they believed were not caused by a physical health condition (e.g. migraine).

As well as a refined selection of the new items, participants in Phase 2 also completed two further measures, as follows.

##### Perseverative Thinking Questionnaire (PTQ; Ehring et al., 2011)

This measure was included to enable the assessment of the new measure’s convergent validity. Without an existing measure of cognitive appraisals of dissociation available for comparison, a measure of proneness to more general ruminative negative thinking was judged to be an appropriate alternative. Indeed, Hunter *et al*. (2003) implicate ‘compulsive self-scrutiny’ (p. 1459) in the maintenance of depersonalisation, suggesting a negative and ruminative quality to the cognitive processes involved in this form of dissociation.

The PTQ is a 15-item scale assessing trait ruminative negative thinking, such as ‘I keep thinking about the same issues all the time’. Items are rated from ‘0, never’ to ‘4, almost always’ and refer to how the respondent ‘*typically* think[s] about negative experiences or problems’. The range of this scale is 0 to 60. Higher scores indicate greater severity of trait negative ruminative thinking.

In the Phase 2 group (n = 8287), the Cronbach’s alpha for the PTQ was 0.97 and model fit for a one-factor 15 item scale structure was adequate for the large sample size [χ^2^ = 8099.36, d.f. = 90, *p* < 0.001, comparative fit index (CFI) = 0.907, Tucker–Lewis index (TLI) = 0.892, root mean square error of approximation (RMSEA) = 0.106, standardised root mean square residual (SRMR) = 0.037].

##### Černis Felt Sense of Anomaly (ČEFSA) scale (Černis et al., submitted)

The ČEFSA is a 35-item scale assessing dissociative experiences taking the form of subjective experiences of strangeness (FSA). This includes items such as ‘I don’t fully experience emotions’ and ‘I feel like a stranger to myself’ rated on a 5-point Likert scale from ‘0, never’ to ‘4 always’. The range of this scale is 0 to 140 and rates the past 2 weeks. Higher scores indicate greater levels of FSA-type dissociative experiences.

Model fit in the Phase 2 group for this scale’s 7-factor 35-item structure was good (χ^2^ = 14831.29, d.f. = 539, *p* < 0.001, CFI = 0.920, TLI = 0.911, RMSEA = 0.057, SRMR = 0.048), with a high Cronbach’s alpha (0.97).

#### Statistical analysis

Analyses were conducted in R, version 3.5.1 (R Core Team, 2018) with packages psych (version 1.8.12; Revelle, 2018) and lavaan (version 0.6-3.1295; Rosseel, 2018). Exploratory factor analyses (EFA) with oblimin rotation and maximum likelihood estimator were carried out to assess the structure of items and refine the item pool by discarding poor-fitting items. Items were judged to fit poorly if they loaded weakly to a factor (less than 0.3), had commonalities less than 0.3, or loaded with similar strength onto multiple factors (loadings within 0.2). Note this latter criterion only applied during Phase 1, as Phase 2 analysis indicated a one-factor structure. The number of factors to extract was determined through parallel analysis and inspection of the scree plot.

The final measure model was assessed using confirmatory factor analysis (CFA) with MLR robust maximum likelihood estimator. The measure’s psychometric properties were also assessed, including a test–re-test reliability statistic, which used the test–re-test phase data collected specifically for this purpose. Convergent validity with the ČEFSA and PTQ were also assessed via Pearson’s correlation, and internal reliability using Cronbach’s alpha. Test–re-test reliability was examined using the intraclass correlation (ICC) between Week 1 and Week 2 data collected specifically for this purpose (test–re-test group).

### Results

#### Measure development

##### Phase 1

Confirming that factor analysis was appropriate for the general population group, Bartlett’s test of sphericity was significant (χ^2^ = 51,166.67, d.f. = 1225, *p* < 0.001) and the Kaiser–Meyer–Olkin test of sampling adequacy was high (KMO = 0.97).

Following the criteria for removing poor-fitting items, and after removing items with inconsistent theoretical content (e.g. appraisals referring to oneself, rather than to dissociative experiences), EFA of Phase 1 data (*n* = 1615) led to 27 of the original 50 items being discarded. Additionally, two items were re-worded at this stage to improve clarity of meaning (shown in Supplementary material): ‘I’m so lonely’ became ‘I am all alone’, and ‘This must mean I’m not human’ was re-worded to ‘This must mean I’m an alien, ghost, or not human’.

##### Phase 2

In Phase 2, a new sample of general population respondents (*n* = 8287) completed the refined pool of 23 items. Confirming that factor analysis was appropriate, Bartlett’s test of sphericity was significant (χ^2^ = 12,3890.00, d.f. = 253, *p* < 0.001) and the Kaiser–Meyer–Olkin test of sampling adequacy was high (KMO = 0.97). This sample was split using R’s random sampling function into a development (*n* = 4143) and validation (*n* = 4144) sample for analysis.

In the development sample, the scree plot and parallel analysis tests indicated that a one-factor structure was the most appropriate fit for the data. Following factor analysis, a further six items were discarded for poor fit, and four items due to high correlated residuals with other items. The resulting 13-item one-factor model explained 52% of the variance.

CFA in the validation sample demonstrated a good fit for this model (χ^2^ = 900.639, d.f. = 65, *p* < 0.001, CFI = 0.962, TLI = 0.955, RMSEA = 0.056, SRMR = 0.025). Factor loadings of the final items in this sample are shown in Table 2.

**Table 2.** Factor loadings for the final items of the measure for the non-clinical and clinical validation groups

		Participant group	
Item		Non-clinical (Phase 2, validation sample) (*n* = 4144)	Clinical (*n* = 1026)
1	I can’t trust my own mind.	0.78	0.69
2	Someone has done something to me.	0.58	0.64
3	Something is terribly wrong.	0.78	0.76
4	I’m losing my mind.	0.82	0.78
5	I’m not really ‘me’.	0.76	0.77
6	I am all alone.	0.71	0.66
7	I don’t look right to other people right now.	0.75	0.71
8	I must be sick.	0.76	0.70
9	I’m not in the same world as everyone else.	0.65	0.73
10	This is because I am evil.	0.55	0.58
11	Now I won’t be able to do the things I wanted.	0.72	0.68
12	It’s not me in control right now.	0.73	0.74
13	This might last forever.	0.73	0.72

#### Measure reliability and validity

The 13-item Cognitive Appraisals of Dissociation in Psychosis (CAD-P) measure had excellent internal consistency (*n* = 4144, Cronbach’s α = 0.93). It was also found to have excellent one-week test–re-test reliability [*n* = 140, ICC = 0.90, 95% confidence interval (CI) = 0.85–0.94, *p* < 0.001].

Furthermore, good convergent validity was found when tested by Pearson’s correlation with the PTQ (Ehring *et al*., 2011) (*n* = 8287, *r* = 0.71, *p* < 0.001), and with the ČEFSA (Černis *et al*., submitted) (*n* = 8287, *r* = 0.81, *p* < 0.001). As there was a significant difference between genders on the PTQ (*p* = 0.023; females: mean = 34.97, *SD* = 13.85; males: mean = 33.85, *SD* = 13.96), convergent validity was also tested for females and males separately. Both indicated high correlation between the CAD-P and PTQ (females: *n* = 7103, *r* = 0.71, *p* < 0.001; males: *n* = 962, *r* = 0.72, *p* < 0.001).

## Part 2: Clinical validation

### Method

#### Procedure and participants

The design was a cross-sectional self-report questionnaire study. The study was supported by the National Institute of Health Research (NIHR) Clinical Research Network (CRN). Participants were recruited by CRN research assistants and clinical studies officers embedded in clinical teams and Research and Development departments across 36 NHS trusts. Inclusion criteria were broad: any person (age 16 years or over), currently under the care of an NHS mental health service, with a diagnosis of non-affective psychosis, who was willing and able to give informed consent to participate. Exclusion criteria were: insufficient English language to complete the questionnaires with support, and an affective psychosis diagnosis (i.e. psychotic depression, bipolar disorder).

Recruitment took place between 18 October 2019 and 19 March 2020. Datasets from 1038 participants were returned. For this analysis, only cases without high levels of missing data in the CAD-P measure (less than or equal to 20% missing) were retained for analysis. This resulted in a participant group of 1026 patients.

In this group, the majority of participants were White (66.96%), male (69.88%) and had a diagnosis of schizophrenia (64.52%). The mean age of the sample was 41.49 years (*SD* = 12.29). See Table 1 for full demographic and descriptive details.

For the second part of the analysis, only cases without high levels of missing data (less than or equal to 20% missing) on the CAD-P, ČEFSA and the two psychosis symptom measures were retained. This resulted in a subgroup of *n* = 1015. The demographic details and descriptive statistics for this subgroup can be found in the Supplementary material.

#### Measures

Participants answered the 13-item version of the CAD-P developed in Part 1 (see Appendix for the full scale), as well as the Perseverative Thinking Questionnaire (Ehring *et al*., 2011) and the Černis Felt Sense of Anomaly (ČEFSA) scale (Černis *et al*., submitted), as described in Part 1. In this group (*n* = 1026) both scales had good internal reliability (ČEFSA: Cronbach’s alpha = 0.97; PTQ: 0.96) and good model fit (ČEFSA: χ^2^ = 1530.378, d.f. = 539, *p* < 0.001, CFI = 0.932, TLI = 0.925, RMSEA = 0.042, SRMR = 0.042; PTQ: χ^2^ = 450.929, d.f. = 90, *p* < 0.001, CFI = 0.949, TLI = 0.941, RMSEA = 0.063, SRMR = 0.029). Participants also completed measures of paranoia and hallucinations, as follows.

##### Revised Green Paranoid Thoughts Scale (R-GPTS Persecution; Freeman et al., 2019)

The R-GPTS is a scale assessing paranoia via ideas of reference and persecution subscales. The persecution subscale of the R-GPTS was used in this study. This subscale consists of ten items (e.g. ‘Certain individuals have had it in for me’), rated over the past month on a 5-point Likert scale from ‘0, not at all’ to ‘4, totally’. Higher scores indicate higher levels of paranoia, with a score above 18 indicating ‘severe’ levels of perceived persecution.

In the clinical group (*n* = 1026), the Cronbach’s alpha for this scale was good (0.92), as was the model fit (χ^2^ = 4007.887, d.f. = 45, *p* < 0.001, CFI = 0.968, TLI = 0.959, RMSEA = 0.060, SRMR = 0.025).

##### Specific Psychotic Experiences Questionnaire (SPEQ-H; Ronald et al., 2013)

The SPEQ consists of four scales which each assess a key psychotic experience. The hallucinations scale (SPEQ-H) was used in this study. This asks respondents to rate how frequently they have recently had particular experiences (e.g. ‘How often do you: hear noises or sounds when there is nothing about to explain them?’) using a 6-point Likert scale (‘0, not at all’ to ‘5, daily’). Higher scores indicate higher levels of hallucinatory experiences. This scale was adapted to include two further items assessing voice hearing: ‘How often do you… hear voices saying words or sentences when there is no one around that might account for it’ and ‘…hear two or more unexplained voices talking to each other’. Indicating that the additional items are consistent with the original items, the Cronbach’s alpha for the adapted SPEQ-H in this group (*n* = 1026) was high (0.93). Model fit for a single-factor scale structure was also adequate for a large sample size (χ^2^ = 711.491, d.f. = 44, *p* < 0.001, CFI = 0.831, TLI = 0.789, RMSEA = 0.122, SRMR = 0.065).

#### Statistical analysis

Analyses were conducted in R, version 3.6.3 (R Core Team, 2020) with packages psych (version 1.9.12.31; Revelle, 2019) and lavaan (version 0.6-5; Rosseel *et al*., 2019).

#### Psychometric validation

The single factor model identified in Part 1 was assessed using CFA with MLR robust maximum likelihood estimator in the clinical subsample (*n* = 1007). Convergent validity of the scale with the ČEFSA and PTQ were assessed via Pearson’s correlation, and its internal reliability via Cronbach’s alpha. Test–re-test data were not collected for this population.

#### Relationship to psychosis variables

Additionally, to begin to demonstrate the value of the CAD-P measure in psychosis research, linear regression models were used to determine whether cognitive appraisals of dissociation explained more variance in psychotic symptoms (paranoia and hallucinations) than dissociative experiences alone.

Data from the subgroup of 1015 participants with low (≤20%) missing data on the CAD-P, ČEFSA, R-GPTS and SPEQ-H were used for this analysis. Missing data for all four scales were imputed using the mice package (version 3.8.0; van Buuren and Groothuis-Oudshoorn, 2020) in order to obtain total scores. Simple and multiple linear regression analyses were then carried out entering the ČEFSA and CAD-P measures as independent variables and the R-GPTS and SPEQ-H measures as dependent variables. As well as inspecting and comparing the estimates of each regression model, models were also formally tested for significant difference using ANOVA analyses.

### Results

#### Psychometric validation

CFA in the clinical group indicated a single factor model had a good fit to the data (χ^2^ = 253.630, d.f. = 65, *p* < 0.001, CFI = 0.959, TLI = 0.950, RMSEA = 0.053, SRMR = 0.029). Factor loadings for the final items in this group are shown in Table 2.

In this sample, the CAD-P had good internal consistency (Cronbach’s α = 0.93) and good convergent validity with the ČEFSA (Černis *et al*., submitted) (*r* = 0.80, *p* < 0.001) and the PTQ (Ehring *et al*., 2011) (*r* = 0.73, *p* < 0.001). As there was a significant difference between genders for the PTQ (*t* = 2.74, *p* = 0.006; females: mean = 32.57, *SD* = 16.98; males: mean = 29.36, *SD* = 16.09), convergent validity between the PTQ and CAD-P was also confirmed for females and males separately (females: *r* = 0.70, *p* < 0.001; males: *r* = 0.75, *p* < 0.001).

#### Relationship to psychosis variables

##### Paranoia

With the R-GPTS Persecution scale as the dependent variable, simple regression analysis with the ČEFSA as the independent variable found that ČEFSA scores explained 34.3% of the variance in R-GPTS scores (β = 0.586, 95% CI: 0.566–0.606, *p* < 0.001). On its own, CAD-P explained 36.4% of the variance in R-GPTS (β = 0.604, 95% CI: 0.559–0.649, *p* < 0.001).

When both ČEFSA and CAD-P were entered as independent variables, the multiple regression model explained 39.6% of the variance in R-GPTS scores (ČEFSA: β = 0.291, 95% CI: 0.260–0.322, *p* < 0.001; CAD-P: β = 0.374, 95% CI: 0.303–0.446, *p* < 0.001): an increase of 5.3% compared with ČEFSA alone. An ANOVA confirmed that this model was a significantly better fit to the data than either simple regression model (*F* = 53.72, d.f. = 1, *p* < 0.001).

##### Hallucinations

A simple linear regression model with SPEQ-H as the dependent variable and ČEFSA as the independent variable explained 40.1% of the variance in SPEQ-H scores (β = 0.634, 95% CI: 0.609–0.659, *p* < 0.001). A simple linear regression model with CAD-P as the independent variable explained 35.0% of the variance (β = 0.593, 95% CI: 0.532–0.653, *p* < 0.001).

Including both variables in a multiple regression model explained 42.4% of variance in SPEQ-H scores (ČEFSA: β = 0.441, 95% CI: 0.401–0.481, *p* < 0.001; CAD-P: β = 0.245, 95% CI: 0.153–0.337, *p* < 0.001): an increase of 2.3% compared with the simple regression model of ČEFSA alone. An ANOVA found that the multiple regression model was a statistically significantly better fit to the data than either simple regression model (*F* = 129.28, d.f. = 1, *p* < 0.001).

### Discussion

To the authors’ knowledge, this study – and its resulting measure – are the first to explicitly address cognitive appraisals of dissociative experiences in the context of psychosis. The measure identifies 13 key cognitive appraisals, all loading onto a single factor. A copy of the full scale can be found in the Appendix. The CAD-P scale has good psychometric properties in both non-clinical and clinical (psychosis) groups: the model-fit is robust, internal reliability is high, and convergent validity with related measures is also good. Where test–re-test reliability was assessed, this was also high for a 1-week interval. When added to regression models, the CAD-P explained additional variance in both paranoia and hallucinations scores in a clinical group, above and beyond that explained by a dissociation measure alone. In general, therefore, the novel scale appears to fulfil the study aim of providing a method for assessing cognitive appraisals relevant to dissociation in psychosis for clinical and research purposes.

Items of the CAD-P, such as ‘I must be sick’ and ‘I’m losing my mind’ are consistent with the proposed similarities between dissociation and anxiety appraisals (Baker *et al*., 2007; Hunter *et al*., 2003; Hunter *et al*., 2014). These appraisals reflect concerns about dissociative experiences being inherently dangerous, or a sign of imminent danger. However, this study also suggests that a number of other appraisals may be important to the experience of dissociation in psychosis. Items reflecting negative beliefs about self (‘This is because I’m evil’) and other people (‘Someone has done something to me’) reflect current understanding of beliefs common in patients experiencing high levels of paranoia (Collett *et al*., 2016; Stopa *et al*., 2013). Further, and of particular note, a number of items indicate concerns about control and ownership over oneself and one’s actions (‘I can’t trust my own mind’, ‘I’m not really “me”’ and ‘It’s not me in control right now’). Whilst maladaptive beliefs about control are a feature of a number of mental health disorders, these statements suggest that experiencing one’s self and internal world as ‘other’ can directly lead people to doubt their autonomy and control. Following Maher (1974), who proposed that within a context of particular beliefs and cognitive biases, anomalous experiences ‘demand explanation’ which may culminate in the adoption of an explanatory delusional belief, it is understandable how the above appraisals might result in delusions of control or passive influence. It is interesting to note that these particular psychotic symptoms have been found to be more common in dissociative identity disorder than in psychosis patients (Laddis and Dell, 2012). These items of the CAD-P, therefore, appear to capture concerns that are specific to the experience of dissociation, but which may be highly relevant to the ‘fuzzy’ boundary between dissociation and psychosis (Renard *et al*., 2017). Further exploration of dissociation – and cognitive appraisals of such experiences – may therefore be particularly appropriate in clinically high-risk (CHR) or prodromal presentations. The broad range of items might be a strength of the CAD-P: clinicians may wish to use the scale initially to enquire further about which appraisals cause the most distress or are the most relevant to their client’s specific circumstances. Subsequent treatment could then be tailored to these appraisals, for example, through the use of behavioural experiments in therapies following a cognitive behavioural approach.

Another strength of the CAD-P is its good psychometric properties in the non-clinical and clinical groups. Regarding convergent validity, it is promising that the CAD-P correlated highly in both samples with the PTQ (Ehring *et al*., 2011). The PTQ measures ruminative negative thinking style, which may be expected to occur as a result of catastrophic cognitive appraisals. Rumination can also be seen as a form of intense internal focus, which Hunter and colleagues (Hunter *et al*., 2003) propose may be common in depersonalisation. The current study determined a correlational relationship between the cognitive appraisals of dissociation and rumination – however, our understanding of dissociation may benefit from research which seeks to determine direction of effect between the two constructs. The CAD-P may be a valuable tool for research such as this in studies using experimental designs, network analysis methods, and treatment outcome (mechanism) studies.

This utility was demonstrated in the current study by using the CAD-P to demonstrate that cognitive appraisals of dissociation explained additional variance in paranoia (persecution) and hallucinations over and above dissociation alone. Interestingly, there were differences between the results for hallucinations and paranoia. For paranoia, cognitive appraisals of dissociation explained approximately the same proportion of variance as FSA-dissociation. However, for hallucinations, cognitive appraisals of dissociation explained closer to half the proportion of variance as did FSA-dissociation. Tentatively, these findings could be interpreted as reflecting the robust relationship between dissociative experience and hallucinations (Longden *et al*., 2020), and the importance of cognition in the development of paranoia (Freeman, 2016). A clinically relevant aim for future research may therefore be to explore the impact on psychotic symptoms of cognitive restructuring for appraisals of dissociation; in particular, whether this may be a mechanism to lessen positive psychotic symptoms. Exploration of these appraisals in relation to negative psychotic symptoms would also be of interest, particularly as many of the CAD-P items contain negative beliefs, or describe passive responses to dissociative experiences.

Limitations of the current study include the manner in which items were developed. As item development drew upon only 12 clinical interviews, it is possible that important cognitions were not elicited and therefore not added to the item pool. For example, cognitive appraisals regarding brain damage or functional impairment have been demonstrated as important in PTSD patients (Samuelson *et al*., 2017), and may also be relevant to dissociation or psychosis.

The sampling method presents another potential limitation of the current study. Whilst providing large sample sizes – a strength of the study that allowed for rigorous analysis – recruitment via Facebook advertisements in Part 1 resulted in a biased sample. The non-clinical samples were drawn from the general population of the UK only, and contained a very high proportion of female and White respondents, with notably high levels of self-reported ongoing mental health difficulties. It is likely that this is the result of self-selection bias due to the title and description of the study sample. Despite the large number of male respondents and respondents from a range of ethnic groups, the participant pool was therefore not representative of the general population. Due to the sampling method, participation was limited to those aged 18 or over. Further validation of the CAD-P in a younger age range would extend the utility of the scale. In particular, this is likely to be important in studies of participants with CHR presentations, where the age range might be expected to include people below the age of 18.

Finally, there are two methodological limitations. Test–re-test data were not collected from the clinical participants in Part 2. As a result, despite the strong test–re-test result in the non-clinical sample (Part 1), this property of the scale remains unknown in clinical contexts and requires further validation. Convergent validity of the CAD-P was assessed using only one dissociation measure. Analysis of validity using a broader range of dissociative measures (particularly the Dissociative Experiences Scale; Carlson and Putnam, 1993) would also be beneficial.

Nonetheless, the CAD-P scale may prove to be a useful tool for researchers and clinicians to identify, assess and investigate cognitive appraisals in individuals with dissociation, especially in the context of psychosis.

## Supplementary material

For supplementary material accompanying this paper visit https://doi.org/10.1017/S1352465820000958. click here to view supplementary material

## Appendix: The Cognitive Appraisals of Dissociation in Psychosis (CAD-P) measure

Please rate how often you think the following when you are feeling strange, disconnected, unreal or ‘dissociated’.

*Please note that this should NOT be whilst under the influence of drugs, alcohol or legal highs.*

**Table:**

		Never	Rarely	Sometimes	Often	Always
1	I can’t trust my own mind.	0	1	2	3	4
2	Someone has done something to me.	0	1	2	3	4
3	Something is terribly wrong.	0	1	2	3	4
4	I’m losing my mind.	0	1	2	3	4
5	I’m not really ‘me’.	0	1	2	3	4
6	I am all alone.	0	1	2	3	4
7	I don’t look right to other people right now.	0	1	2	3	4
8	I must be sick.	0	1	2	3	4
9	I’m not in the same world as everyone else.	0	1	2	3	4
10	This is because I am evil.	0	1	2	3	4
11	Now I won’t be able to do the things I wanted.	0	1	2	3	4
12	It’s not me in control right now.	0	1	2	3	4
13	This might last forever.	0	1	2	3	4

*Scoring*: sum score of all items.

*Note that*: raw scores cannot distinguish between many appraisals occurring infrequently, and a small number of appraisals experienced very frequently. In clinical contexts, therefore, further interpretation of responses may be required.
