# Supplementary material for: Cognitive appraisals of dissociation in psychosis: a new brief measure
Source: Behav Cogn Psychother. 2020 Dec 28;49(4):472–84. doi: 10.1017/S1352465820000958 (PMC8293624; doi:10.1017/S1352465820000958)
Supplement: Supplementary file 1 [file S1352465820000958sup001.docx]

**Supplementary Material 1: Full item pool tested in Phase 1**

Changes subsequently made to two items (no. 32 & 48) are shown after the arrow (🡪).

| 1 | The world is trying to bring me down | 26 | I'm losing my mind |
| --- | --- | --- | --- |
| 2 | This might last forever | 27 | I'm not really "me" |
| 3 | This might be a dream | 28 | Here we go again |
| 4 | Everyone's going to notice this | 29 | I don't want this |
| 5 | I can't trust my own mind | 30 | I can't cope with this |
| 6 | Too much is happening right now | 31 | People are going to think I'm mad |
| 7 | Someone has done something to me | 32 | I'm so lonely 🡪 I am all alone |
| 8 | I don't care any more | 33 | I'm just tired |
| 9 | I'm pathetic | 34 | Reality has changed |
| 10 | I'm in a computer simulation | 35 | There's no point fighting this |
| 11 | I could get stuck like this | 36 | I am worthless |
| 12 | I just need to wait for it to pass | 37 | I don't look right to other people right now |
| 13 | People are going to judge me for this | 38 | I must be sick |
| 14 | This feeling is dangerous | 39 | I'm not in the same world as everyone else |
| 15 | This is too difficult | 40 | I can't do this any more |
| 16 | There's a hidden meaning in this feeling | 41 | This is because I am evil |
| 17 | This proves I am unloveable | 42 | I'm just getting confused |
| 18 | Something is terribly wrong | 43 | I must be special |
| 19 | This is because there is something wrong with me | 44 | Now I won't be able to do the things I wanted |
| 20 | I must be exhausted | 45 | It's not me in control right now |
| 21 | My life is in danger | 46 | People are going to think I'm being rude |
| 22 | This proves I am abnormal | 47 | This could spiral out of control |
| 23 | I want this to stop now | 48 | This must mean I'm not human 🡪 This must mean I’m an alien, ghost, or not human |
| 24 | I'm being stupid | 49 | It's ok - it's just one of those things |
| 25 | This shows I am weak | 50 | This is too much |

**Supplementary Material 2: Additional demographics and descriptive statistics**

| **Table s1. Showing the demographic data and descriptive statistics for the clinical subgroup used in the regression analyses (n=1015)** | | | | | | | | | |
| --- | --- | --- | --- | --- | --- | --- | --- | --- | --- |
| **Demographic** | | | **Mean (Standard Deviation)** | | | | **Range** | | |
| **Age** | | | | 41.44 (12.29) | | | 18 - 74 | | |
| **Demographic** | |  | | | | | **n (% of group)** | | |
| **Gender** | | | | | *Female:*  *Male:*  *Other:* | | 297 (29.26%)  709 (69.85%)  5 (0.49%) | | |
| **Ethnicity** | | | | *White (any):*  *Mixed / Multiple:*  *Asian (any):*  *Black (any):*  *Other:* | | | 681 (67.09%)  43 (4.24%)  96 (9.46%)  172 (16.95%)  17 (1.67%) | | |
| **Diagnosis** | *Schizophrenia*  *Schizoaffective*  *Delusional Disorder*  *Psychotic Disorder NOS**  *First Episode Psychosis*  *Other Schizophrenia Spectrum Disorder* | | | | | | 657 (64.73%)  149 (14.68%)  14 (1.38%)  68 (6.70%)  104 (10.25%)  23 (2.27%) | | |
| **Measure** | | | | | | **Group Mean (SD)** | | **Range** | **Test for gender differences** |
| *Cognitive Appraisals of Dissociation in Psychosis* | | | | | 18.86 (13.22) | | | 0 - 52 | *Females*: 20.00 (13.86)  *Males*: 18.36 (12.89)  *t-test*: p=0.082 *non sig*. |
| *Černis Felt Sense of Anomaly scale* | | | | | 40.54 (30.39) | | | 0 - 140 | *Females*: 40.97 (31.42)  *Males*: 40.25 (29.93)  *t-test*: p=0.737 *non sig*. |
| *Revised Green Paranoid Thoughts Scale*  *(Persecution subscale)* | | | | | | 14.06 (12.10) | | 0 - 40 | *Females*: 14.00 (12.25)  *Males*: 13.94 (11.98)  *t-test*: p=0.947 *non sig*. |
| *Specific Psychotic Experiences Questionnaire (Hallucinations subscale)* | | | | | | 17.11 (15.99) | | 0 - 55 | *Females*: 18.27 (16.62)  *Males*: 16.61 (15.65)  *t-test*: p=0.143 *non sig*. |
